# A quick and selected overview of the expert panel on effective ways of investing in health

**DOI:** 10.1186/s13690-017-0219-3

**Published:** 2017-12-04

**Authors:** Pedro Pita Barros

**Affiliations:** Nova School of Business and Economics, Universidade Nova de Lisboa, Campus de Campolide, 1099-032 Lisbon, Portugal

**Keywords:** Expert panel, Policy discussion

## Abstract

The European Commission created the Expert Panel on Effective Ways of Investing in Health (EXPH) in 2012. The EXPH started its activities in July 2013 and ended its first term in May 2016. A personal review of the Expert Panel contributions in its first term is provided.

## The panel and how it worked

The European Commission created the Expert Panel on Effective Ways of Investing in Health (EXPH) in 2012, after an open call for applications. The EXPH started its activities in July 2013 and ended its first term in May 2016. The objective of the European Commission with setting up this Expert Panel was to receive advice on health sector-related issues.

The work of the EXPH is available publicly in documents (“Opinions”). These Opinions intend to convey the view of the members of the panel on the questions included in the mandates agreed with the European Commission. Each Opinion is originated by a mandate set between the European Commission and the Expert Panel.

The 12 members of the panel distributed themselves into working groups dealing with each mandate. The final version was discussed and approved in plenary sessions of the panel. Working groups could, and did in several cases, include participation of external experts and have members of the European Commission as observers during the work sessions.

This brief description of how the panel worked helps to understand how the topics treated in the different Opinions were defined and how the interaction with the European Commission was set. The Opinions reflect only the panel’s views, obviously, and do not intend to be official documents of the European Commission. They were written in a way to make them of use to the wide community interested in the organization and evolution of the health sector in Europe. The EXPH produced ten opinions during its first term (see list in Appendix).

The curious reader can obtain further details from the website of the EXPH (https://ec.europa.eu/health/expert_panel/home_en).

## What results and impact did the expert panel have?

The impact, or contribution, of the EXPH is not easy to assess. As an expert panel, its role was not to provide reviews of existing literature on the several topics addressed. Its role was to provide advice based on the best knowledge available to the EXPH members. Often, it was possible to trace the relevant literature or evidence, but not always. Sometimes, “knowledge” results from incorporation of information from many different sources over time. The Opinions reflect the balance of the different training and visions of the members. The richness of diversity is a contribution to the wide discussion of the matters included in the Opinions.

At a more general level, advice can result in actions being taken or being deterred. It is rare to find occasions of direct links from advice (especially, advice made public) to decisions by institutions or individuals. Moreover, the result of (useful) advice may take years to produce visible result, in particular when new policy initiatives emerge from such advice. In this sense, there is no clear measurement of the impact of the EXPH.

The Opinions produced provide an examination of topics of relevance to policy making in the health sector in several countries. They provide a common framework to discuss policy options. Although the Opinions are not scientific research published in academic journals, the matters discussed are of interest to health economics, public health, and health services research. As examples of potential impact, Bayle et al. [2] and Astier-Peña et al. [1], in their analysis of the Spanish health system, do make reference to the PPP Opinion [3] and to the Patient Safety Opinion [5], respectively.

From the ten Opinions produced by the Expert Panel, I briefly report on three of them. These were selected to illustrate the different origins of the mandates of the Expert Panel: reflections based on prior work (the PPP opinion), providing an updated view on a permanently relevant issue (the primary care opinion), and opening ground for new discussions (the disruptive innovation opinion). The remaining documents that were produced during the first term of the Expert Panel fall into one of those types. They all address issues of policy interest, written having in mind how scientific knowledge can be made useful for policy makers.

### The public-private partnerships opinion

The Public-Private Partnerships (PPP) Opinion [3] was the first mandate of the EXPH to become publicly available. The mandate by the European Commission consisted of several specific questions concerning a DG SANCO report externally commissioned on PPPs in Europe. This Opinion, unlike the ones that followed, was reactive to a previously existing document. The EXPH expressed a common view on the scarcity and quality of publicly available data and evidence about PPPs in the health sector. An implication of the little public disclosure of information on PPPs is the difficulty in gathering scientific evidence on the cost-effectiveness of PPPs in comparison with traditional forms of public financed and managed provision of care. Moreover, the Opinion made explicit the view that PPPs are a tool to achieve health system objectives and not a goal per se. This view implies, among others, that full service PPPs (those that include both infrastructure and management of clinical activities) should be similar to public alternatives of care from the point of view of patient experience in terms of access and out-of-pocket payments. Thus, PPPs can be a useful instrument in the toolbox of health-system managers though they are not an all-purpose solution, and have their own problems. AS clearly stated “a PPP cannot be good if the underlying public investment decision is not a good one, no matter how well designed is the contract between the public partner and the private partner.” ([3], p.4).

An important policy question asked in the mandate by the European Commission related to the prospects of using EU structural funds to develop PPPs in health care. The answer to this question in the Opinion was that, with the present state of knowledge, it is advisable to avoid such a policy move.

Whenever these, and other, observations by the EXPH become part of the discussion process of new public-private partnership projects, a useful, yet difficult to measure, contribution exists.

### The primary care opinion

Another Opinion with a strong impact is the primary care Opinion [4]. As example, this Opinion, and its definition of primary care, received attention from *The Lancet* (2014), bringing it to a much wider audience and positive comments were informally directed to the panel members.

This Opinion started with a clear request: “provide a comprehensive and operational definition of primary care – which includes goals, functions and players involved” ([4], p. 7).

There are other elements in the mandate and in the final Opinion, though the definition is clearly a central element with wide potential impact. The goals for health systems that are in the background of the definition of primary care are those set by the World Health Organization (WHO): health improvement, more responsiveness to expectations of the population, and financial fairness (including elements both of health insurance and adequate redistribution of the financial burden of health care).

In line with the mandate, a core definition of primary care was proposed: “The Expert Panel considers primary care as the provision of universally accessible, integrated person-centred, comprehensive health and community services provided by a team of professionals accountable for addressing a large majority of personal health needs. These services are delivered in a sustainable partnership with patients and informal caregivers, in the context of family and community, and play a central role on the overall coordination and continuity of people’s care.

“The professionals active in primary care teams include, among others, dentists, dieticians, general pratictioners / family physicians, midwives, nurses, occupational therapists, optometrists, pharmacists, physiotherapists, psychologists and social workers.” ([4], p. 18).

The definition sets primary care as a team effort, in which coordination of different providers is a key issue. This coordination is becoming more important as populations age and the number of comorbidities at older ages increases. Primary care also has challenges related to access, given that it is the natural main and first point of contact of people with the health system when feeling ill. The definition introduces a more active and explicit role for patients and informal caregivers.

The acceptance of this definition by the health sector participants has long-run implications for health systems design and workforce training. It does not have an immediate impact on policy making. It is intended to help the transformation and evolution of health systems in a direction that better answers to people’s needs, expectations, and available resources.

The definition encapsulates a full plan of action to health systems. The point of universal access and financial protection of the population is in line with the WHO pledge for universal coverage. Primary care systems need to consider how the formal provision of care interacts with partners, patients, and informal caregivers, in a long-term relationship. This moves away from the usual passive role of patients and their informal caregivers (gaining importance as longevity increases the very old population with chronic conditions that wants to live a normal life in their place of residence). Also partnerships and coordination across health care professionals requires different workforce training and relationships. For many health systems, coping with this definition will lead to important changes in the way primary care is provided to the population.

### The disruptive innovation opinion

The Opinion on Disruptive Innovation [6] had a different starting point from the other Opinions by the EXPH. The mandate reflected the novelty of the concept of disruptive innovation (coined in 1995 by Clayton Christensen). Disruptive innovation has become a buzzword, synonym of high-tech devices. It has also started to be applied in the health care sector.

A contribution from this Opinion is precisely the definition of disruptive innovation in health care, as it will mean a different role than in other sectors: “The Expert Panel understands ‘disruptive innovation’ in health care as a type of innovation that creates new networks and new organizations based on a new set of values, involving new players, which makes it possible to improve health outcomes and other valuable goals, such as equity and efficiency. This innovation displaces older systems and ways of doing things” ([6], p. 23).

This definition looks at disruptive innovation as a changer of culture and structure of organizations. It is not a simple aspect of some new technology becoming available. It needs to transform significantly the “old way of doing things”. As such, disruptive innovation will be mostly unpredictable. From a policy perspective, there is no clear policy measure that can be adopted that will lead with certainty to a “disruptive innovation”. Instead, policies aimed at promoting disruptive innovations in health care should focus on enabling facilitating factors and removing barriers to the emergence of disruptive innovations. Mechanisms to foster disruptive innovation should make it easier to experiment with new models of health care provision, accepting failure as a part of the process. The institutional framework set by health systems needs to be able to cope with the cultural and structural changes that may occur following a disruptive innovation, such as decommissioning old structures that become obsolete, retraining the workforce, or change the culture of users of health care, can face strong resistance resulting from habits or due to vested interests.

This definition also makes clear that high technology content is neither necessary nor sufficient to have disruptive innovations in health care. An innovation with low technology content but able to transform the culture and the way a service is provided will be disruptive, while a more technology-intensive innovation that retains the same culture and organization is not. The very same technology may or may not be part of a disruptive innovation. Take electronic health records, for example. If they just improve care provided to patients, then it is a continuing innovation. It is a valuable innovation though not a disruptive one. But if the technology for the electronic health record changes the “old ways” and creates a new culture and leads to new organizations to provide health care, then it becomes disruptive.

### Other opinions from the expert panel

Several other opinions touch upon important issues: cross-border health care cooperation, competition amongst health care providers, and a proposal of framework to assess health care reforms. These Opinions address important policy aspects. The legislation (Directive) on cross-border health care services was promoted by the European Commission. Obstacles appear to be greater than expected, in particular lack of information and the different interpretations of the Directive. The Opinion suggests possible steps that would help cross-border health services to improve people’s health while not disorganizing health systems.

Competition amongst health care providers has often been seen as “magic wand” to solve problems. It is often coupled with freedom of choice as a patient’s right. The Opinion clarifies that analysis of health systems can and should decouple patient’s rights from competition among health care providers, even though these are often closely connected, as competition allows more choice. The central message of the Opinion is simple: competition amongst health care providers should be regarded as a tool for achieving health systems goals. As such, it will be useful in some circumstances but not in others. No general presumption about it being good or bad for people’s health can be established, as the details will matter. Still, it is unlikely that a single instrument (competition among health care providers) is able to reach the several goals that health systems have. Thus, competition must be assessed in its contribution to several goals and whether other instruments should accompany it.

The Opinion on a framework to evaluate reform effects provides a general discussion on the process of health reforms, including political coherence and feasibility. It also proposes a simple template to highlight the main effects to always consider when proposing health care reforms. It is not an automatic algorithm to determine the outcome (there is no such tool). It works as a check list on the impacts in the usual goals of health systems and it can be easily applied.

## Conclusions

The Expert Panel on Effective Ways of Investing in Health was created to provide advice to the European Commission. The public dissemination of the work produced does allow for a wider impact. The mandates received by the Expert Panel had a focus on broad and conceptual issues. Thus, there is no direct, immediate, and visible impact on policies at the European level arising from the existence of this panel.

The ideas as recommendations expressed in the Expert Panel Opinions will make their way to policies in the background, shaping the debates that later lead to policy actions. Of course, sometimes “wrong” policy actions may be avoided by a proper policy discussion. This type of impact is even more difficult to identify. Several Opinions went through a process of public consultation. The public consultations received hundreds of comments, revealing the interest of many stakeholders that was raised by the Expert Panel work.

Overall, the Expert Panel brought consolidated knowledge, benefiting from diversity of backgrounds and experiences of panel members. It translated to the policy debate arena a set of frameworks and ideas that will be a useful conceptual umbrella under which concrete policy options will be discussed, adopted and implemented to improve people’s lives.

## Appendix

### List of opinions

1. Health and Economic Analysis for an Evaluation of the Public-Private Partnerships in Health Care Delivery across Europe
2. Definition and Endorsement of Criteria to Identify Priority Areas When Assessing the Performance of Health Systems
3. Definition of a frame of reference in relation to primary care with a special emphasis on financing systems and referral systems
4. Future EU Agenda on quality of health care with a special emphasis on patient safety
5. Competition among health care providers in the European Union - Investigating policy options
6. Cross-border Cooperation
7. Access to health services in the European Union
8. Disruptive Innovation. Considerations for health and health care in Europe
9. Best practices and potential pitfalls in public health sector commissioning from private providers
10. Typology of health policy reforms and framework for evaluating reform effects

## References

[1] Astier-Peña MP, Torijano-Casalengira ML, Olivera-Cañadas G, Silvestre-Busto C, Agra-Varela Y, Maderuela-Fernandez JA (2015). Are Spanish primary care professionals aware of patient safety?. Eur J Pub Health.

[2] Bayle MS, Ruiz SF (2014). Movilizaciones sociales e profesionales en España frenet a la contrarreforma sanitaria. Saúde em Debate.

[3] EXPH – Expert Panel on Effective Ways of Investing in Health (2014). Health and economic analysis for an evaluation of the public-private partnerships in health care delivery across Europe, February 2014.

[4] EXPH – Expert Panel on Effective Ways of Investing in Health (2014). Report on definition of a frame of reference in relation to primary care with a special emphasis on financing systems and referral systems, 10 July 2014.

[5] EXPH – Expert Panel on Effective Ways of Investing in Health (2014). Final report on future EU agenda on quality of health care with a special emphasis on patient safety, 9 October 2014.

[6] EXPH – Expert Panel on Effective Ways of Investing in Health (2016). Report on disruptive innovation, 29 February 2016.

